# Supplementary material for: RNA inhibitors of nuclear proteins responsible for multiple organ dysfunction syndrome
Source: Nat Commun. 2019 Jan 10;10:116. doi: 10.1038/s41467-018-08030-y (PMC6328615; doi:10.1038/s41467-018-08030-y)
Supplement: Supplementary file 2 — Supplementary Information [file 41467_2018_8030_MOESM2_ESM.pdf]

Supplementary Information

RNA inhibitors of nuclear proteins responsible for multiple organ dysfunction syndrome.

Urak et al.

Supplementary Methods

Platelet Aggregation assay.

Washed platelets were prepared as described in the Methods section. Platelets were re-suspended in Tyrode buffer to a final concentration of 2.5×10^8 platelets per mL. For the platelet aggregation studies, 400 µl of washed platelets were stirred at 37 °C with either collagen (1 µg mL^{-1}), histone H4 (10 µg mL^{-1}), selected RNA rounds (15 µg mL^{-1}) in a cuvette of an aggregometer (Chrono-log Model 560-VS) and light transmittance was recorded. Aggregation was measured as percent change in light transmission, where 100 % refers to transmittance through blank solution.

TLR activation studies.

EA.Hy926 cells were seeded in a 96-well flat bottom plate at a density of 15,000 cells per well in 100 µl of media. After 24 h the medium was removed and replaced with 100 µl of Opti-MEM containing either calf thymus histones (CTH) at varying concentrations or LPS (50 µg mL^{-1}) with or without Aptamers KU7 and KU9 (50 µg mL^{-1}). After 24 h, the supernatant was collected, processed and quantified according to manufacturer's protocol in the human interleukine-6 (IL-6) Quantikine® ELISA kit (R&D Systems, Minneapolis, MN).

Double-filter binding assay.

Double-filter nitrocellulose binding assay was preformed to determine the binding affinity (K_D) of the aptamers for their target. Synthetic aptamers (KU7 and KU9) were radiolabeled with gamma-^{32}P-ATP using T4 polynucleotide kinase (PNK) as follows: A total reaction volume of 20 µL was made up of 20 pmol of aptamer RNA, 2 µL PNK (New England Biolabs, Ipswich, MA), 2 µL PNK reaction buffer (New England Biolabs, Ipswich, MA), 2 µL gamma-^{32}P-ATP (PerkinElmer, Waltham, MA), and diH_2O. The mixture was incubated at 37 °C for 30 min and 65 °C for 20 min to heat inactivate the PNK. 20 µL of 1× BB was added to the reaction followed

by a centrifugation step through a G25 purification column (GE Healthcare, Little Chalfont, United Kingdom, IL) according to manufactures instructions. Labeling efficiency was determined by a scintillation counter. All radiolabeled RNAs were diluted in 1× BB to 2,000 cpm per mL. 5 µL of 2,000 cpm per mL radiolabeled RNA was incubated at 37 °C for 5, 50, and 100 mins respectively with 15 µL of either human histone H4 serially diluted in 1× BB. The binding reactions were loaded onto a dot blot apparatus (composed of nitrocellulose membrane on the top, nylon membrane (Sigma-Aldrich, St. Louis, MO) in the middle and Whatman paper (Sigma-Aldrich, St. Louis, MO) on the bottom). Treatment of the nitrocellulose membrane was as follows: pretreated with 0.5 M KOH (Sigma-Aldrich, St. Louis, MO) for 20 min, quick wash with diH_2O, and equilibrated in 0.1 M Tris HCl 7.5 for 45 min, washed with diH_2O, and transferred to 1× BB for 20 min before l (Sigma-Aldrich, St. Louis, MO). The nylon was also incubated in 1× BB for 20 min before being loaded on the manifold. Before loading the RNA/protein samples, the wells were washed with 100 µL of 1× BB. The amount of RNA bound (nitrocellulose) versus unbound (nylon) was determined by densitometry of imaged membrane on a Fuji Phosphor imager.

Supplementary Figure 1

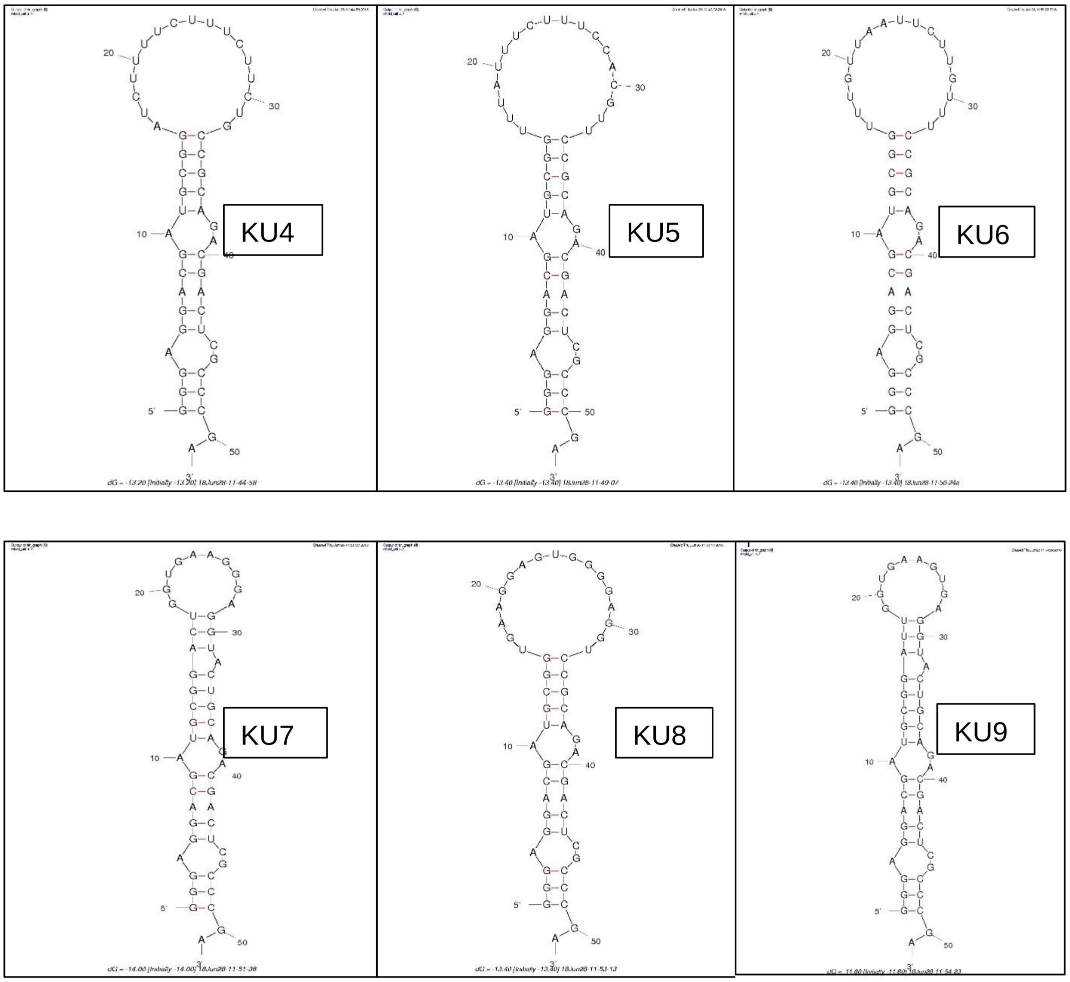

Supplementary Figure 1. Theoretical secondary RNA structures for the top 6 histone aptamers.

Secondary RNA structures for histone aptamers KU4 – KU9 predicted using Mfold.

Supplementary Figure 2

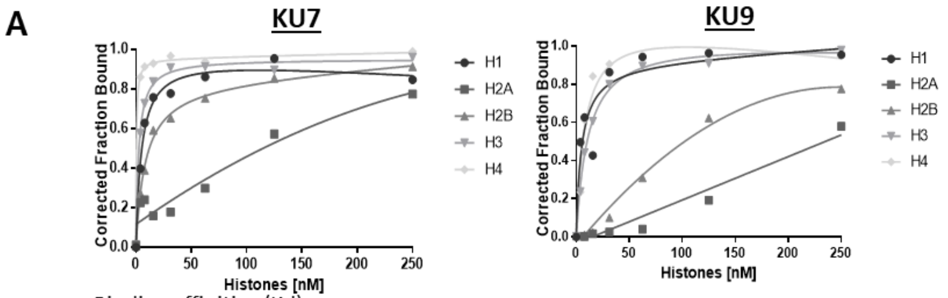

Binding affinities (Kd)

	KU7 (nM)	KU9 (nM)
H4	0.4	7.6
H3	2.5	11.2
H2B	8.4	NA
H2A	NA	NA
H1	5	5.3

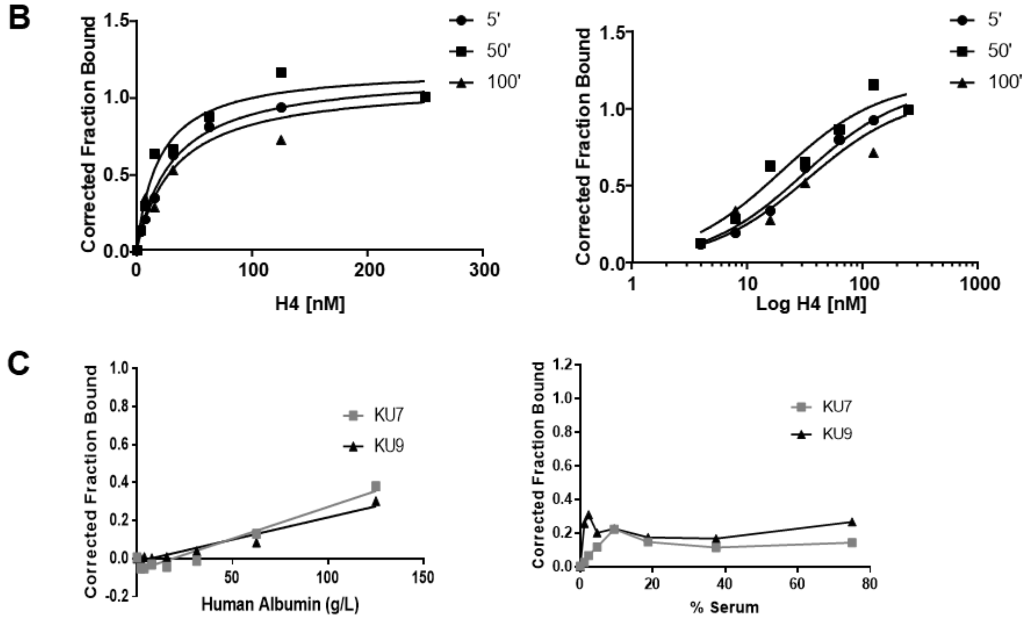

Supplementary Figure 2. Sensitivity and specificity for selected RNA aptamers. **A)** Binding of KU7 (left panel) and KU9 (right panel) to recombinant histones H1, H2A, H2B, H3 and H4. Binding constants are listed in table (bottom). **B)** Binding of KU7 to H4 histones (middle, left and right panel) at 5, 50 and 100 mins to determine equilibrium binding time. **C)** Binding of selected RNA aptamers KU7 and KU9 human serum albumin (lower, left panel) and to serum (lower, right panel).

Supplementary Figure 3

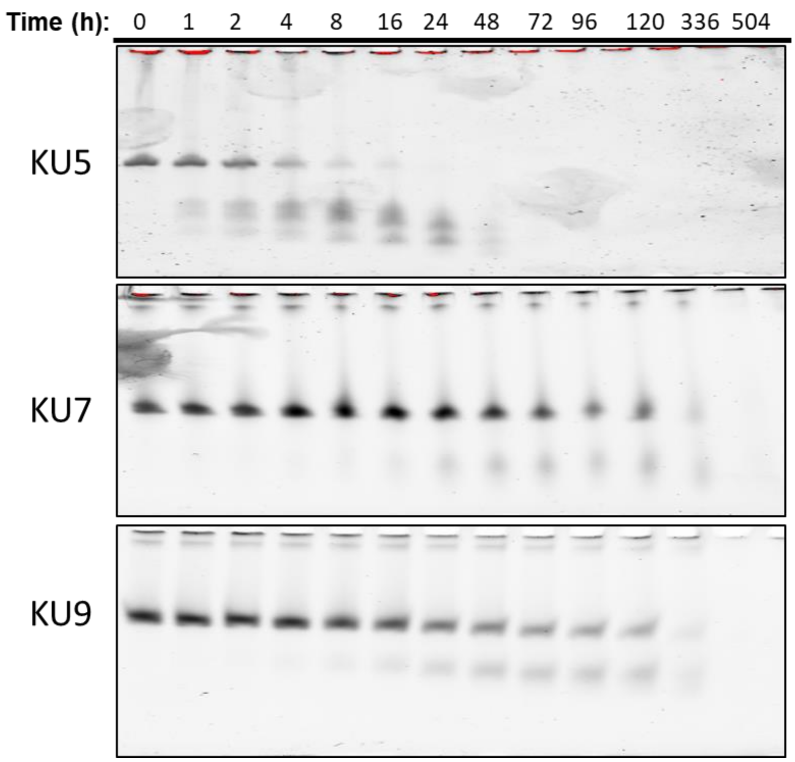

Supplementary Figure 3. Stability measurements of individual histone RNA aptamer sequences

Full-sized 8M urea gels of the aptamers highlighting the degradation or stability of each aptamer.

Supplementary Figure 4

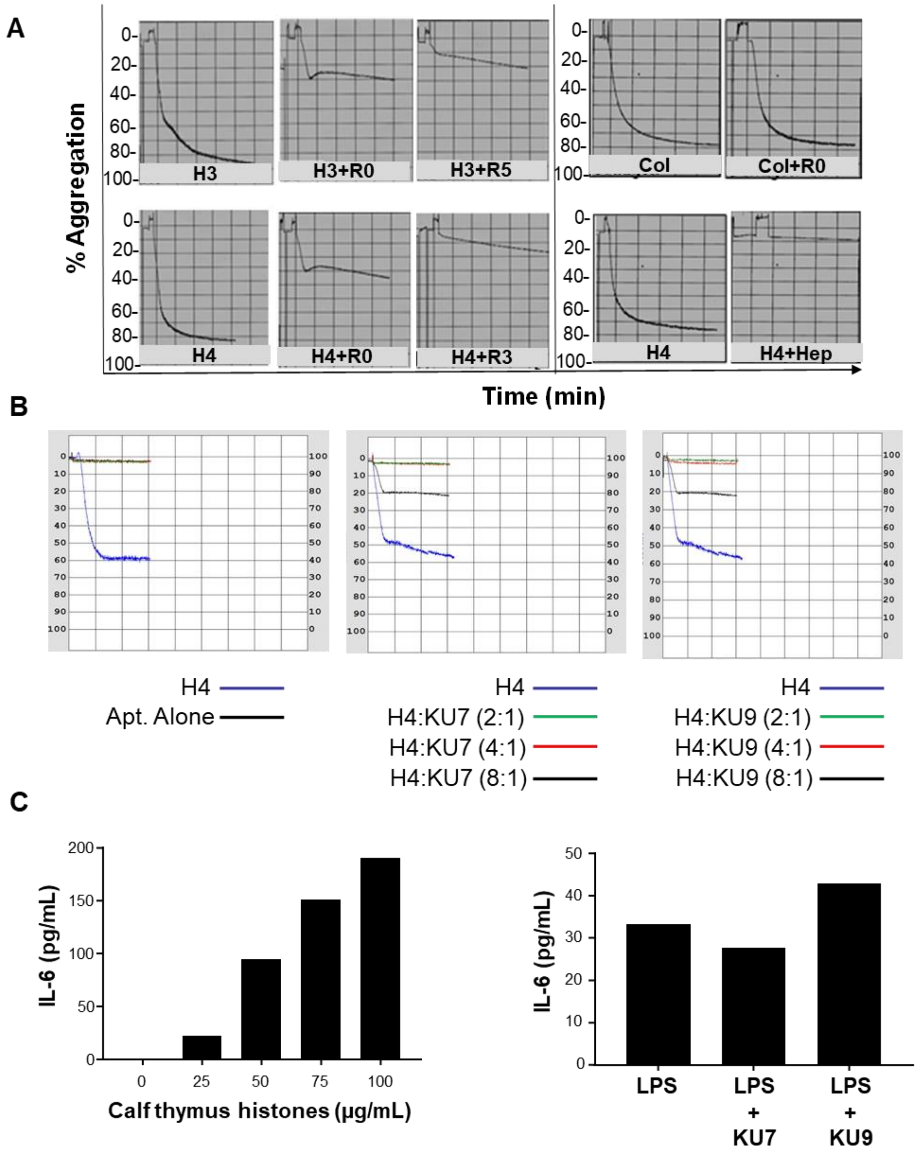

Supplementary Figure 4. *In vitro* efficacy of RNA aptamers. A) Human platelet aggregation. Collagen **(**Col), human histones (H3 and H4), Round 0 RNA (R0), Round 3 RNA (R3), Round 5 RNA (R5), heparin (Hep). Platelets derived from 3 independent healthy donors. **B)** Platelets were treated with 10 µg/mL of histone H4 alone (blue) or 10 µg/mL aptamers alone (black). Platelets were treated with 10 µg/mL of histone H4 alone (blue) or histone plus varying amounts of RNA aptamer KU7 (middle panel) or KU9 (right panel). **C)** TLR activation. IL-6 protein levels in supernatants of EA.Hy926 cells treated with various amounts (0 to 100 µg/mL) of CTH (left panel) or LPS (50 µg mL^{-1}) plus aptamers KU7 or KU9 (50 µg mL^{-1}).

Supplementary Figure 5

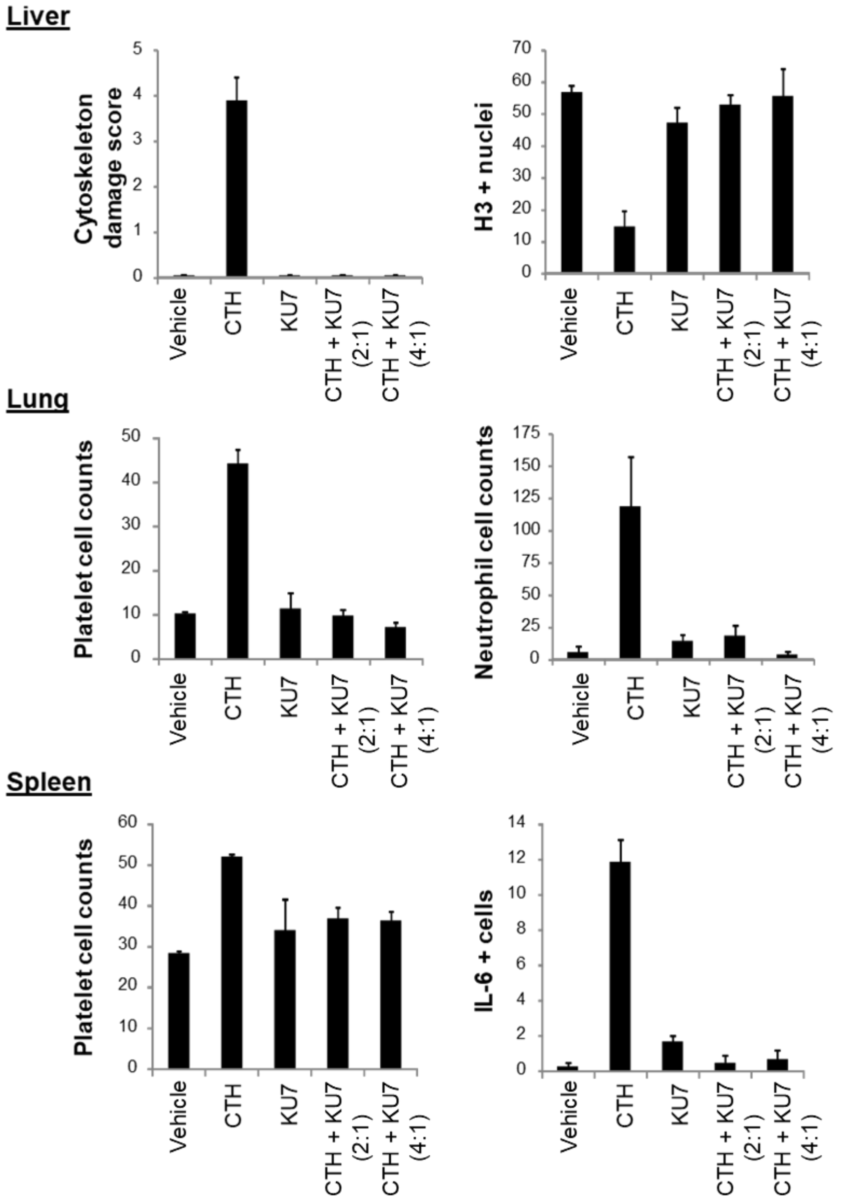

Supplementary Figure 5. Efficacy of histone aptamer in murine model of MODS. Quantitative analyses of immunostaining of mouse liver (top panels), lung (middle panels) and spleen (bottom panels) tissues. Bars represent mean values of 2-4 mice (each mouse estimated from 4-5 independent confocal images taken at 20× magnification with 2× zoom, using the image analysis program, NIH ImageJ). Cytoskeleton damage score (liver) was assigned a score from 0 to 5 (no damage = 0, most severe = 5). Platelet cell counts (lung and spleen) represent mean optical density measurements. Error bars = standard deviation of 2-4 mice (each mouse estimated from 4-5 independent confocal images).

Supplementary Table 1. Histone selection protocol

	Round	Ratio of Protein:RNA	RNA (pmol)	Protein (pmol)	[Protein] (nM)	Binding Reaction Volume (mL)	Binding Time	[NaCl] (mM)	Pre-Clear conditions	Pre-Clear Time	Washes (10mL)
Positive Selection (Histones) and Negative Selection (hIgG and BSA)	1	1:10	1000	100	10	10	10 min in solution<br>10 min with disk	150	hIgG	20 min in solution<br>20 min with disk	3 x 5 min
	2	1:10	1000	100	10	10	5 min in solution<br>5 min with disk	150	hIgG	20 min in solution<br>20 min with disk	3 x 10 min
	3	1:20	1000	50	5	10	5 min in solution<br>5 min with disk	150	hIgG	20 min in solution<br>20 min with disk	3 x 10 min
	4	1:40	2000	50	5	10	5 min in solution<br>5 min with disk	150	hIgG + BSA	10 min in solution<br>10 min with disk	3 x 10 min
	5	1:40	2000	25	5	10	5 min in solution<br>5 min with disk	150	hIgG + BSA	10 min in solution<br>3 x 10 min with disk	3 x 15 min
	6	1:80	2000	25	5	5	5 min in solution<br>5 min with disk	150	hIgG + BSA	10 min in solution<br>10 min with disk	3 x 30 min
Negative Selection (hIgG and BSA)	7	-	-	-	-	-	-	150	hIgG + BSA	10 min in solution<br>3 x 10 min with disk	-
	8	-	-	-	-	-	-	150	hIgG + BSA	10 min in solution<br>3 x 10 min with disk	-

Supplementary Table 2. Affinity measurements

	Histone H3 (nM)	Histone H4 (nM)	CTH (nM)	Human Albumin	Human Serum
**Round 0**	4.221	1.963	-		
**Round 8**	1.306	1.038	-	N/A	N/A
**KU7**	0.3117	0.68	4.109	N/A	N/A
**KU9**	5.239	0.8575	12.46	N/A	N/A

Supplementary Table 3. Top aptamer sequences from histone selection

Original name	name	sequence	Fold enrichment (R3:5)	Fold enrichment (6:8)
0081_H4	KU4	GGGAGGACGAUGCGGAUCUUUUCUUUCUUCUGCCGCAGACGACUCGCCCGA	9.704	0.47
0065_H4	KU5	GGGAGGACGAUGCGGUUUAUUUCUUUCCACGUUCCGCAGACGACUCGCCCGA	9.699	0.47
0120_H4	KU6	GGGAGGACGAUGCGGUUUGUUAAUUCUUGUUUCCGCAGACGACUCGCCCGA	9.22	0.23
0145_H32	KU7	GGGAGGACGAUGCGGACUGGUGAAGGGAGGUACUGCAGACGACUCGCCCGA	6.91	2.22
0236_H32	KU8	GGGAGGACGAUGCGGUGAAGGAGUGGGGAGGUCCGCAGACGACUCGCCCGA	6.74	1.3
0243_H32	KU9	GGGAGGACGAUGCGGAUUGGUGAAGUGAGGUACUGCAGACGACUCGCCCGA	6.58	0.6

Supplementary Table 4. Next-generation sequencing (NGS) primers

Illumina primers with Barcode	sequence
BC01	AAT GAT ACG GCG ACC ACC GAG ATC TAC ACT CTT TCC CTA CAC GAC GCT CTT CCG ATC TAA GAA GAA GGG AGG ACG ATG CGG
BC02	AAT GAT ACG GCG ACC ACC GAG ATC TAC ACT CTT TCC CTA CAC GAC GCT CTT CCG ATC TGG CAA GAA GGG AGG ACG ATG CGG
BC03	AAT GAT ACG GCG ACC ACC GAG ATC TAC ACT CTT TCC CTA CAC GAC GCT CTT CCG ATC TCC TAA GAA GGG AGG ACG ATG CGG
BC04	AAT GAT ACG GCG ACC ACC GAG ATC TAC ACT CTT TCC CTA CAC GAC GCT CTT CCG ATC TCG AGA GAA GGG AGG ACG ATG CGG
BC05	AAT GAT ACG GCG ACC ACC GAG ATC TAC ACT CTT TCC CTA CAC GAC GCT CTT CCG ATC TGT GGA GAA GGG AGG ACG ATG CGG
BC06	AAT GAT ACG GCG ACC ACC GAG ATC TAC ACT CTT TCC CTA CAC GAC GCT CTT CCG ATC TTA CGA GAA GGG AGG ACG ATG CGG
BC07	AAT GAT ACG GCG ACC ACC GAG ATC TAC ACT CTT TCC CTA CAC GAC GCT CTT CCG ATC TAT CCA GAA GGG AGG ACG ATG CGG
